# Assay validation and interspecific comparison of salivary glucocorticoids in three amphibian species

**DOI:** 10.1093/conphys/coy055

**Published:** 2018-09-27

**Authors:** Talisin T Hammond, Zoe A Au, Allison C Hartman, Corinne L Richards-Zawacki

**Affiliations:** Department of Biological Sciences, University of Pittsburgh, 105 Clapp Hall, 5th Ave. at Ruskin Ave., Pittsburgh, PA, USA

**Keywords:** ACTH, handling, non-invasive, saliva, stress

## Abstract

Amphibians are one of the most threatened groups of species, facing stressors ranging from habitat degradation and pollution to disease and overexploitation. Stress hormones (glucocorticoids, GCs) provide one quantitative metric of stress, and developing non-invasive methods for measuring GCs in amphibians would clarify how diverse environmental stressors impact individual health in this taxonomic group. Saliva is an advantageous matrix for quantifying GCs, as it is sampled less invasively than plasma while still detecting both baseline and acute elevation of GCs within a short timeframe. Little work has employed this method in amphibian species, and it has never been pharmacologically and biologically validated. Here, we conduct analytical, pharmacological and biological validation experiments for measuring salivary corticosterone in three amphibian species: the American bullfrog (*Rana catesbeiana*), the green frog (*Rana clamitans*) and the northern leopard frog (*Rana pipiens*). These species are faced with a broad range of environmental challenges, and in part of its range *R. pipiens* populations are currently in decline. In addition to demonstrating that this method can be reliably used in multiple amphibian species, we present an examination of intrinsic biological factors (sex, body condition) that may contribute to GC secretion, and a demonstration that saliva can be collected from free-living animals in the field to quantify corticosterone. Our findings suggest that saliva may be useful for less invasively quantifying GCs in many amphibian species.

## Teaser

Amphibians, as the most imperiled vertebrate group, are currently threatened by stressors including habitat loss, disease and pollution. Methods for reliably and non-invasively measuring stress hormones can elucidate the impact of stressors on amphibian health. This study validates a new method for measuring salivary stress hormones in three amphibian species.

## Introduction

As one of the most threatened taxonomic groups, amphibians are currently faced with a broad range of stressors including habitat loss, disease, overexploitation and pollution (Hof *et al.*, 2011; Vié *et al.*, 2009). Stress hormones (glucocorticoids, GCs) can provide a quantitative measure of the physiological impacts of these stressors, and may facilitate a better understanding of how environmental change and disease affect amphibian health. Specific relationships between GCs and fitness are variable and context-dependent, and recent work has demonstrated the importance of considering life-history variables and individual condition when attempting to explain these relationships (Jaatinen *et al.*, 2013; Vitousek *et al.*, 2018). Still, there are often ties between GCs and individual or population health and fitness (Hing *et al.*, 2016; Vitousek *et al.*, 2018), and in a broad range of taxonomic groups, less invasive measures of GCs (e.g. feces, urine, saliva) have allowed for physiological monitoring of free-living populations while avoiding unnecessary disturbance of vulnerable species (Millspaugh and Washburn, 2004; Wikelski and Cooke, 2006). The small body size of many amphibians can also prohibit repeated plasma sampling on the same individual, thus, it is desirable to develop less invasive methods to facilitate repeated measures of GCs from one individual. Despite the imperiled status of many amphibian species, few non-invasive methods for quantifying GCs have been validated in this group (Narayan, 2013).

Some non-invasive methods for quantifying GCs have been validated in amphibian species. Urinary corticosterone is the most commonly used tool, though fecal sampling has also been employed (Narayan, 2013). Both methods usually require holding animals for longer periods (hours to days) in order to detect a stress response. Waterborne GCs provide a valuable, completely non-invasive option that shows good parallelism with free plasma corticosterone (Gabor *et al.*, 2013, 2016), however, this method measures GC release rate rather than GC concentration, and in some cases doing so can prevent high-resolution distinction between baseline and acutely elevated GCs (e.g. Gabor *et al.*, 2013, but see Reedy *et al.*, 2014). This could in part be because animals are required to sit in a water bath for ~60 min for the baseline sample collection, and acute GC elevation often begins within minutes (Sapolsky *et al.*, 2000). Skin secretions provide another potentially useful, novel tool for quantifying GCs, though this method has only been tested in a small number of individuals (Santymire *et al.*, 2018).

Saliva is a valuable substrate for measuring both baseline and acutely elevated GCs: it is less invasively sampled than plasma and, unlike plasma and fecal samples, it often requires little to no sample preparation and extraction prior to assay (Vining *et al.*, 1986). Moreover, unlike feces and urine, which usually reveal GC responses to acute stressors after many hours or days post-stress, saliva sampling allows for detection of both baseline and acutely increased GCs within minutes, as GCs in saliva generally increase along similar timelines to plasma (Sheriff *et al.*, 2011). Salivary GCs are often stable at room temperature for multiple days and usually reliably track plasma free GCs, which are generally thought to be the biologically active portion of circulating GCs (Hofman, 2001; Malisch and Breuner, 2010; Sheriff *et al.*, 2011). To date, few studies have used saliva to quantify GCs in amphibians (Cayuela *et al.*, 2017; Janin *et al.*, 2012; Troïanowski *et al.*, 2017), and to our knowledge no method for measuring GCs in saliva has ever been thoroughly validated in this group (Touma and Palme, 2005).

Here, we conduct analytical (spike and recovery, linearity, and parallelism tests), pharmacological (adrenocorticotrophic hormone challenge) and biological (handling challenge) validation experiments to show that saliva samples can be assayed by enzyme immunoassay to reliably reflect baseline and acutely elevated corticosterone levels in three amphibian species: the American bullfrog (*Rana catesbeiana*), the green frog (*Rana clamitans*) and the northern leopard frog (*Rana pipiens*). In addition to testing whether saliva is a useful new matrix for quantifying GCs in amphibians, our results test for effects of sex and body condition on GCs. Our results also demonstrate that this method can be employed in the field in free-living individuals of multiple amphibian species, suggesting that this technique may be broadly useful to practitioners hoping to use less invasive methods to quantify stress in amphibians.

## Methods

### Study sites and species

*Rana catesbeiana* and *R. clamitans* individuals were captured at two sites near the University of Pittsburgh’s Pymatuning Laboratory of Ecology in Linesville, PA. Immediately upon capture, a saliva sample was collected from each individual (see Saliva collection and treatment), snout-vent length (SVL) and mass were measured, and sex and reproductive status were recorded. Individuals were then transported to animal facilities at the University of Pittsburgh, where they were housed individually in plastic tanks and given ~3 weeks to habituate to captivity before being exposed to an ACTH challenge (see ACTH challenge), followed 2 weeks later by a handling challenge (see Handling challenge). *Rana pipiens* individuals were also captured in the wild in Pennsylvania and Vermont and had been in captivity for ~6 months at the time of the study, therefore, saliva samples were not able to be collected from these individuals in the field, however, experimental timelines were otherwise the same. A small number (*n* = 5) of samples were collected from field-captured *R. pipiens* in late April of 2018 and were assayed to provide a point of comparison for this species. All procedures were approved by the University of Pittsburgh’s Animal Care and Use Committee.

### Saliva collection and treatment

Saliva was collected using a Salivette swab (Salimetrics SalivaBio Infant’s Swab). Each swab was cut into four pieces, with one piece used per sample. Within 90 s of initial handling, each focal individual’s mouth was gently opened using a sterile cotton-tipped dry swab (Medical Wire & Equipment Co.) or a sterile pipette tip, and sterile forceps were used to hold the Salivette in the focal individual’s opened mouth for one minute. The saliva-soaked Salivette was then placed in a micro-centrifuge tube and frozen at −20°C for storage and to increase the precipitation of mucins (Toone *et al.*, 2013), which can cause assay interference. Prior to assaying, the swab was removed from the freezer, placed above a plastic filter in its micro-centrifuge tube, and centrifuged for 10 min at 7000 rpm to extract liquid (resulting volumes were generally 30–75 μl). A known volume of saliva (whenever possible 50 μl, but always between 20 and 50 μl) was then transferred to a 0.2 ml micro-centrifuge tube and treated with trichloroacetic acid (TCA; 10 μl TCA per 50 μl saliva). TCA was used because initial testing revealed significant assay interference, likely due to salivary proteins. TCA has been used in other biological samples to precipitate proteins (Cascalheira *et al.*, 2008). After adding TCA, samples were vortexed for ~10 s, incubated for 15 min at room temperature, vortexed again for 10 s, centrifuged for 8 min at 6000 RPM, and a known volume of the supernatant was collected, diluted in assay buffer, and immediately assayed. Previous research has shown that micro-injuries of the mouth causing leakage of blood into the oral mucosa do not impact salivary GCs (Kivlighan *et al.*, 2004), however, samples obviously contaminated with blood (pink or red in coloration, ~7% of samples) were not included in analyses.

### ACTH challenge

To determine whether changes in circulating GCs can be detected in saliva samples, individuals (*N* = 6 females (F), 3 males (M) *R. catesbeiana*, 5F/4M *R. clamitans*, 4F/4M *R. pipiens*) were injected with adrenocorticotrophic hormone (ACTH; Sigma Aldrich A0298, 250 μg dissolved in 2 ml 0.9% saline solution), a commonly used pharmacological stressor, at a dose of 0.45 μg ACTH/g body mass, resulting in injection volumes from ~100 to 400 μl. This dosage has been used in previous studies of amphibians (Graham *et al.*, 2013; Narayan *et al.*, 2011; Touma and Palme, 2005). To control for potential circadian and seasonal changes in corticosterone, all individuals were injected between 1000 and 1400 h in late August (*R. catesbeiana* and *R. clamitans*) or early October (*R. pipiens*; all dates are outside of the active breeding period for these species) of 2017. Individuals were not fed the day before the ACTH challenge to prevent any effects of eating on salivary hormone concentrations. For all individuals, saliva samples were collected immediately prior to injection and at 15, 30 and 60 min, and at 2, 4 and 8 h post-injection. Initial testing revealed that samples at 4 and 8 h after injection were similar, thus, we did not assay samples at 8 h for the majority of individuals. Collecting a baseline (0 min) sample allowed for within-individual comparisons of stressed and unstressed samples.

### Handling challenge

To quantify the stress response to a more biologically relevant stressor, individuals (*N* = 6F/5M *R. catesbeiana*, 7F/6M *R. clamitans*, 6F/7M *R. pipiens*) were removed from their cages and briefly handled (~60 s) before being returned to their cages. Samples were otherwise collected and analyzed exactly as described for the ACTH challenge (see ACTH challenge). Collecting a baseline (0 min) sample allowed for within-individual comparisons of stressed and unstressed samples.

### Enzyme immunoassay

Because corticosterone is the primary GC in most amphibians (Narayan, 2013) we used a competitive enzyme linked immunosorbent assay for corticosterone (R&D Systems KGE009) to quantify GCs in saliva. To analytically validate the assay, spike and recovery, linearity, and parallelism experiments were conducted. Pooled samples for each species were spiked with a known volume of corticosterone standard (250 ng/ml) and assayed at three serial dilutions, and the resulting concentrations were compared to control (assay buffer) spiked samples and un-spiked samples from the same pool. Separate pooled samples for each species were also serially diluted 2-fold (from 1:1 to 1:32) and assayed to establish linearity and parallelism with the standard curve. The manufacturer reported sensitivity (minimum detectable dose) of the assay was 0.028 ng corticosterone/ml, and intra-assay and inter-assay coefficients of variation (CV) were ~6.1 and 6.2%, respectively, though intra-assay CV calculations based on our samples were ~14%.

### Statistical analyses

All statistical analyses were conducted in R (R Core Team, 2017). Spike and recovery was calculated by subtracting concentrations of un-spiked samples from paired spiked samples and dividing by a control (assay buffer) spiked sample of matched concentration. Linearity was assessed for 2-fold serial dilutions by dividing each sample’s concentration by the halved concentration of the previous step’s dilution. Spike and recovery and linearity values within ~80–120% are generally considered suitable (per manufacturer guidelines of assay kit). Parallelism was assessed using an analysis of covariance to test for the significance of an interaction between concentration and type of sample (standard vs. pooled samples; non-significant interactions indicated that samples and the standard curve exhibited parallel slopes).

To test for the effects of ACTH and handling on corticosterone, generalized linear mixed models (GLMMs) were implemented in the lme4 package (Bates *et al.*, 2014) with time (minutes after stressor) and the square of time (to account for the fact that the relationship between time and corticosterone was expected to be inverse-u-shaped and non-zero centered) included as fixed effects. This method allows us to test the prediction that, within each individual, GCs increased over time before decreasing towards baseline. Sex and a measure of body condition (mass divided by SVL) were also included as fixed effects, and individual identity was included as a random effect. Separate models were created for each species’ ACTH and handling challenges. The Satterthwaite approximation (implemented in the lmerTest package) was used to estimate degrees of freedom and to test significance of each fixed effect (Kuznetsova *et al.*, 2015). For each species, Wilcoxon rank sum tests were used to test for differences in GC concentrations between ACTH-treated and handled individuals at baseline (0 min) vs. 30 min post-stress, the time at which most individuals’ GCs peaked. Corticosterone concentrations were log-transformed prior to all analyses.

## Results

### Analytical validation

**Figure 1:**
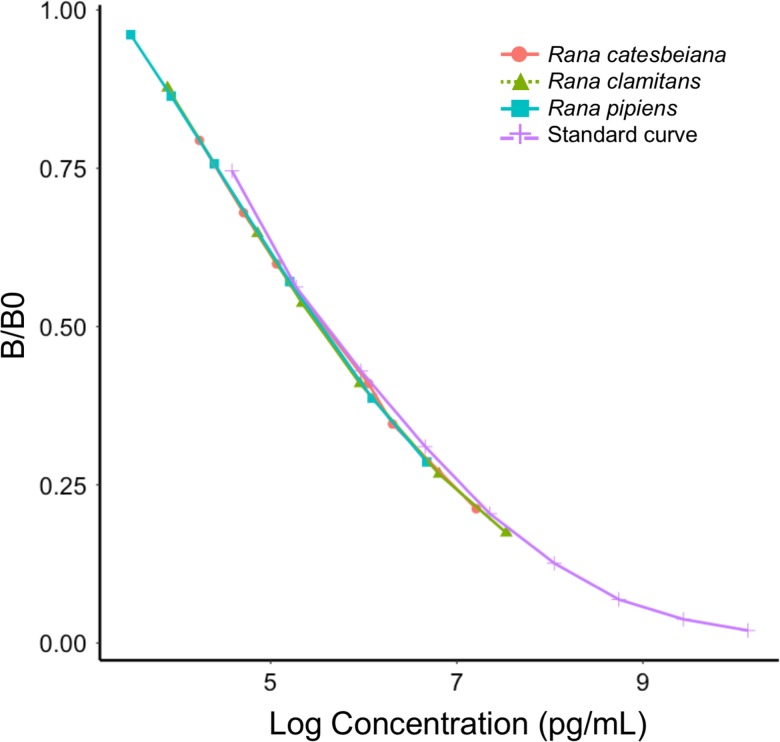
Parallelism between the standard curve and serial dilutions of pooled samples from the focal species. The slope of the standard curve (purple crosses) did not significantly differ from that of 2-fold serial dilutions (1:1–1:32) from *R. catesbeiana* (pink circles), *R. clamitans* (green triangles) or *R. pipiens* (blue squares) samples

Adequate spike and recovery values (*R. catesbeiana*: 81.7%; *R. clamitans*: 87.0%; *R. pipiens*: 77.8%) and linearity values (*R. catesbeiana*: 115%; *R. clamitans*: 98%; *R. pipiens*: 108%) were obtained for all species. Parallelism tests revealed that the slope of serially diluted pooled samples from each species did not differ significantly from the slope of the linear portion of the standard curve (Table S1; Fig. 1).

### ACTH challenge

**Figure 2:**
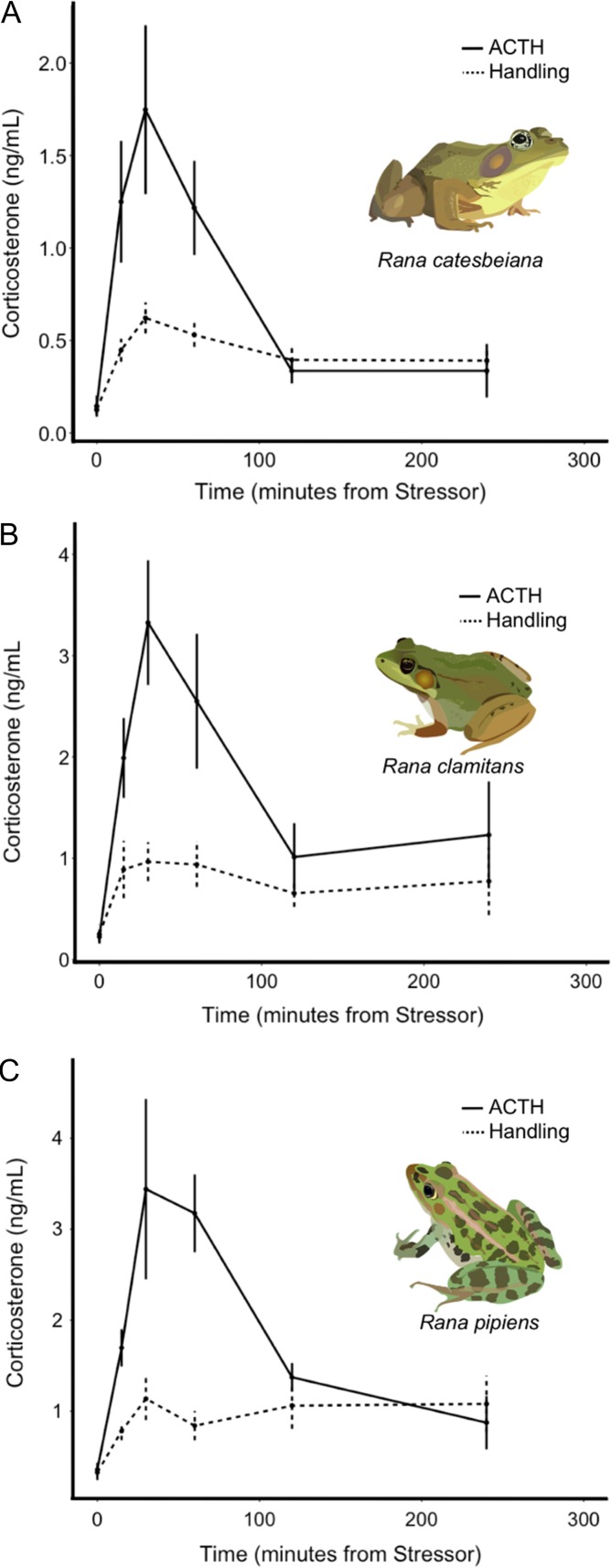
Salivary corticosterone levels as a function of time in response to stress. Strong responses to ACTH (solid line) and handling (dotted line) were documented for *R. catesbeiana* (**A**), *R. clamitans* (**B**) and *R. pipiens* (**C**)

ACTH injection led to clear and significant increases in corticosterone levels over time for all three species (Table 1 and Fig. 2). Corticosterone tended to peak 30 min after injection (Fig. 2). Sex and body condition did not explain significant variation in response to ACTH for any species (Table 1).

**Table 1:** Final, model averaged GLMMs testing for change in corticosterone levels over time in response to ACTH injection for *R. catesbeiana* (A), *R. clamitans* (B) and *R. pipiens* (C). Significant terms are bolded. The significance of the ‘Time’ and/or ‘Time2’ terms indicates a significant change in GCs over time, supporting the effects of ACTH on salivary GCs

*A. Rana catesbeiana*					
Coefficient	Estimate	S.E.	df	*t*-Value	*P*-value
**(Intercept)**	**0.75**	**0.40**	**13.72**	**1.90**	**0.08**
Time	0.71	0.39	32.59	1.83	0.08
**(Time**^**2**^)	**−1.0**	**0.34**	**32.60**	**−2.96**	**0.006**
Sex (M)	−0.31	0.49	4.85	−0.62	0.56
Body condition	0.28	0.27	5.53	1.02	0.35
Random effect	Variance	S.D.			
ID	0.17	0.41			

*B. Rana clamitans*					
Coefficient	Estimate	S.E.	df	*t*-value	*P*-value
**(Intercept)**	**1.00**	**0.39**	**9.29**	**2.58**	**0.03**
**Time**	**0.78**	**0.30**	**37.35**	**2.66**	**0.01**
**Time**^**2**^	**−0.92**	**0.26**	**37.21**	**−3.49**	**0.001**
Sex (M)	−0.11	0.51	6.02	−0.22	0.83
Body condition	−0.05	0.27	6.00	−0.19	0.86
Random effect	Variance	S.D.			
ID	0.43	0.66			

*C. Rana pipiens*					
Coefficient	Estimate	S.E.	df	*t*-value	*P*-value
**(Intercept)**	**1.23**	**0.28**	**38**	**4.43**	**7.8e-05**
**Time**	**0.56**	**0.21**	**38**	**2.66**	**0.01**
**Time**^**2**^	**−0.71**	**0.17**	**38**	**−4.15**	**0.0002**
Sex	−0.27	0.37	38	−0.72	0.47
Body condition	−0.04	0.16	38	−0.28	0.78
Random effect	Variance	S.D.			
ID	0.0	0.0			

### Handling challenge

Handling led to clear and significant increases in corticosterone for all species, with the expected lower magnitude increases than ACTH-induced changes (Wilcoxon rank sum tests with false discovery rate adjusted *P*-values comparing corticosterone concentrations at 30 min for ACTH vs. handling challenges, *R. catesbeiana*: W = 87, *P* = 0.02; *R. clamitans*: W = 100, *P* = 0.01; *R. pipiens*: W = 57, *P* = 0.02; at 0 min all *P*-values testing for differences in salivary corticosterone between ACTH vs. handling challenges are >0.5; Table 2; Fig. 2). Paralleling the ACTH challenge, corticosterone tended to peak ~30 min after the stressor (Fig. 2). *Rana catesbeiana* individuals in worse body condition tended to have to have higher stress-induced corticosterone levels, but no biological factors explained significant variation in *R. clamitans*’ or *R. pipiens*’ response to handling (Table 2).

**Table 2:** Final, model-averaged GLMMs testing for change in corticosterone levels over time in response to handling for *R. catesbeiana* (A), *R. clamitans* (B) and *R. pipiens* (C). Significant terms are bolded. The significance of the ‘Time’ and/or ‘Time2’ terms indicates a significant change in GCs over time, supporting the effects of handling on salivary GCs

*A. Rana catesbeiana*					
Coefficient	Estimate	S.E.	df	*t*-Value	*P*-value
(Intercept)	0.15	0.16	66	0.98	0.33
**Time**	**0.43**	**0.20**	**66**	**2.11**	**0.04**
**(Time**^**2**^)	**−0.39**	**0.17**	**66**	**−2.28**	**0.03**
Sex (M)	−0.03	0.19	66	−0.15	0.88
**Body condition**	**−0.20**	**0.09**	**66**	**−2.31**	**0.02**
Random effect	Variance	S.D.			
ID	1e-19	3.2e-10			

*B. Rana clamitans*					
Coefficient	Estimate	S.E.	df	*t*-Value	*P*-value
(Intercept)	−0.18	0.26	11.59	−0.67	0.52
**Time**	**0.48**	**0.21**	**52.51**	**2.29**	**0.03**
**Time**^**2**^	**−0.53**	**0.19**	**52.13**	**−2.80**	**0.007**
Sex (M)	0.45	0.36	8.16	1.25	0.25
Body condition	−0.14	0.18	8.06	−0.75	0.47
Random effect	Variance	S.D.			
ID	0.30	0.55			

*C. Rana pipiens*					
Coefficient	Estimate	S.E.	df	*t*-Value	*P*-value
(Intercept)	−0.05	0.21	17.93	−0.23	0.82
**Time**	**0.48**	**0.16**	**53.11**	**3.04**	**0.004**
**Time**^**2**^	**−0.44**	**0.13**	**52.5**	**−3.41**	**0.001**
Sex (M)	0.48	0.26	7.98	1.85	0.10
Body condition	−0.22	0.14	7.85	−1.56	0.16
Random effect	Variance	S.D.			
ID	0.03	0.18			

### Baseline corticosterone in captive and free-living animals

**Figure 3:**
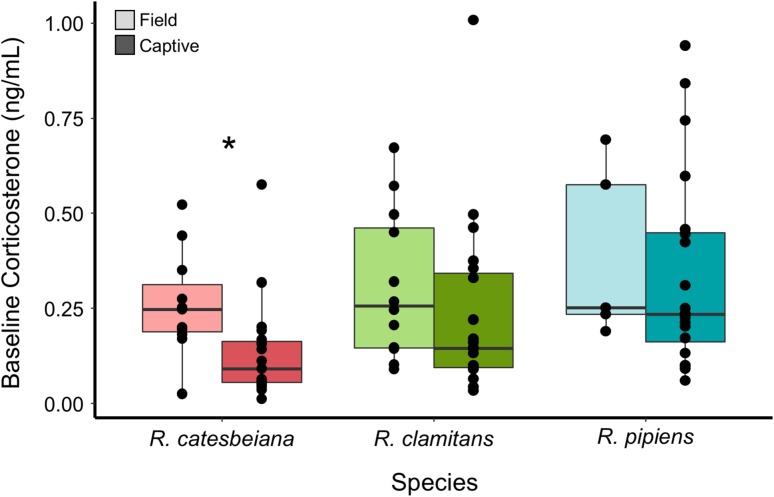
Field and captive baseline salivary corticosterone levels**.** Box and whiskers plots showing baseline salivary corticosterone in the field (dark) and in captivity (light) for *R. catesbeiana* (left), *R. clamitans* (middle) and *R. pipiens* (right). A star indicates that the intraspecific comparison was significant (*P* < 0.05)

Animals of all species exhibited a pattern of higher baseline salivary corticosterone in the field compared to the lab, however, this comparison was only statistically significant for *R. catesbeiana* (Wilcoxon rank sum tests with false discovery rate adjusted *P*-values, *R. catesbeiana*: W = 91, *P* = 0.03; *R. clamitans*: W = 80, *P* = 0.4; *R. pipiens*: W = 39.5, *P* = 0.4; Fig. 3). Sex was predictive of baseline corticosterone for *R. pipiens*, with males exhibiting higher corticosterone levels (Table S2). Sex was not predictive for *R. catesbeiana* or *R. clamitans*, and body condition was not explanatory for any species (Table S2).

## Discussion

Our results show that saliva samples can reliably reflect expected changes in baseline and stress-induced corticosterone levels in three amphibian species. GCs are often used as physiological proxies in studies monitoring individual or population health, as chronically elevated GCs can be induced by anthropogenic changes to environments and are sometimes associated with reduced fitness (Bonier *et al.*, 2009). Despite the imperiled status of amphibians, fewer non-invasive methods for measuring stress have been validated for this taxon as compared to others, with urinary GCs being the most commonly employed non-invasive metric (Narayan, 2013; Touma and Palme, 2005). Saliva may be a particularly advantageous sample matrix, as it is readily available and can be collected within a short (~30 min), catch-and-release timeline to measure both baseline and acutely elevated corticosterone. Quantifying the stress response usually requires either the use of more invasive blood sampling, or holding animals for a longer period (hours to days) to collect fecal or urinary samples. Both of these constraints can be logistically difficult or impossible when studying animals of conservation concern. Waterborne GCs provide another useful measure of GC release rate that is even less invasive than saliva sampling and shows good parallelism with plasma corticosterone (Gabor *et al.*, 2016). One shortcoming of this validation study is that we were unable to collect plasma samples, preventing a comparison of plasma and salivary corticosterone. In other species, salivary GCs generally increase along a similar (within <5–10 min delay) timeline as plasma GCs and show parallelism with free plasma GCs, the more biologically active portion of circulating GCs (Gozansky *et al.*, 2005; Hernandez *et al.*, 2014; Kerlik *et al.*, 2010; Malisch and Breuner, 2010; Teruhisa *et al.*, 1981; Vincent and Michell, 1992; Vining *et al.*, 1983).

When quantifying GCs in novel substances or species, it is critical to analytically, pharmacologically and biologically validate a specific assay for the detection of the parameter in question (Narayan, 2013; Touma and Palme, 2005). Future studies measuring salivary corticosterone in novel amphibian species must include the necessary validation experiments, but our results provide a streamlined protocol for doing so. Most notably, our initial attempts to assay saliva suggested significant interference, likely from salivary proteins; future studies can avoid this pitfall by freezing and centrifuging samples and using TCA pre-treatment to precipitate mucins and other salivary proteins prior to assaying. There are also other challenges associated with using saliva to quantify GCs. First, depending on the concentrations of corticosterone, this method is less likely to work in individuals that are less than ~20 g, and in species with drier mouths. However, preliminary results suggest that adding a step in which the saliva swab is washed may improve hormone recovery, allowing for assaying of smaller volume or low concentration samples. Second, time since last meal, recent activity and other lifestyle factors can alter salivary GCs (Garde *et al.*, 2009; Gibson *et al.*, 1999). While this presents a source of error and increased variation, particularly for samples collected from free-living individuals, such factors are known to impact corticosterone in other bodily fluids, including in plasma (Brandenberger and Follenius, 1975; Legler *et al.*, 1982). Whenever possible, these factors should be controlled and recorded. Timing is also critical: salivary corticosterone levels were already 2–3.5 times (on average) higher than baseline samples as soon as 15 min after initiation of handling; in other species levels increase within <5 min, therefore, baseline samples should be collected as quickly as possible.

Sex and body condition did not explain significant variation in ACTH-induced GCs for any of the focal species. In response to handling, *R. catesbeiana* individuals with lower body condition exhibited elevated GCs, but the magnitude of this effect was limited, and no intrinsic biological factors were explanatory for *R. clamitans* or *R. pipiens*. Baseline corticosterone was higher in *R. pipiens* males in comparison to females, though sex had no relationship with baseline corticosterone for the other two species. Body condition was not predictive of baseline corticosterone. While some reptiles and amphibians do show sex differences in corticosterone responsivity, it is also not unusual for the sexes to exhibit similar corticosterone levels, particularly outside of the breeding season (Moore and Jessop, 2003). Baseline salivary corticosterone showed a cross-species pattern of being higher in the field than in captivity, although this pattern was significant only for *R. catesbeiana*. An individual’s baseline and stressed GCs are context-dependent and frequently differ in captive and field settings (Calisi and Bentley, 2009). With saliva sampling, this difference could also be explained by our inability to control feeding times for animals in the field; in other studies, salivary GCs increase after eating (Garde *et al.*, 2009; Gibson *et al.*, 1999).

While the focal species are not of immediate conservation concern (Hammerson *et al.*, 2004; IUCN, 2015a,b), *R. pipiens* is declining in certain parts of its range, and all three congeners face a broad range of anthropogenic changes, including habitat loss and pollution, mainly from agricultural development (Lannoo, 2005; Rorabaugh, 2005). Disease is another threat facing these ranids: populations of the three focal species are infected with the fungal pathogen *Batrachochytrium dendrobatidis* (*Bd*), which has decimated many amphibian populations globally (Lenker *et al.*, 2014; Skerratt *et al.*, 2007). In general, positive relationships between *Bd* infection and GCs in amphibians have been documented (Gabor *et al.*, 2015; Murone *et al.*, 2016), and future studies of these species may combine salivary corticosterone measurements with *Bd* testing to further examine ties between stress and disease. More generally, our results suggest that this method can work in a variety of species and may be useful to any researcher interested in quantifying amphibian stress responses while limiting their impacts on study individuals.

## Supplementary Material

Supplementary Data
