# Supplementary material for: Assay validation and interspecific comparison of salivary glucocorticoids in three amphibian species
Source: Conserv Physiol. 2018 Sep 27;6(1):coy055. doi: 10.1093/conphys/coy055 (PMC6158758; doi:10.1093/conphys/coy055)
Supplement: Supplementary Data [file coy055_supplementary_tables.docx]

**SUPPLEMENTARY TABLES**

**Table S1. Analysis of covariance results demonstrating that there is no significant difference between the slopes of the standard curve and serial dilutions of pooled samples for any of the focal species.**

1. *R. catesbeiana*

|  | | Df | | S.S. | | M.S | F value | | p-value | |  | |
| --- | --- | --- | --- | --- | --- | --- | --- | --- | --- | --- | --- | --- |
| Concentration | | 1 | | 0.43 | | 0.43 | 800.38 | | 1.8e-08 | |  | |
| Type (*standard or pooled sample*) | | 1 | | 0.0007 | | 0.0007 | 1.34 | | 0.29 | |  | |
| Concentration*Type | | 1 | | 0.0 | | 0.0 | 0.08 | | 0.79 | |  | |
| Residuals | | 7 | | 0.004 | | 0.0005 |  | |  | |  | |
|  | |  | |  | |  |  | |  | |  | |
| 1. *R. clamitans* |  | |  | |  | | |  | |  | |  |
|  | | Df | | S.S. | | M.S | F value | | p-value | |  | |
| Concentration | | 1 | | 0.51 | | 0.51 | 541.41 | | 6.9e-08 | |  | |
| Type (*standard or pooled sample*) | | 1 | | 0.0003 | | 0.0003 | 0.35 | | 0.57 | |  | |
| Concentration*Type | | 1 | | 0.0 | | 0.0 | 0.002 | | 0.96 | |  | |
| Residuals | | 7 | | 0.007 | | 0.0009 |  | |  | |  | |
|  | |  | |  | |  |  | |  | |  | |

1. *R. pipiens*

|  | Df | S.S. | M.S | F value | p-value |  |
| --- | --- | --- | --- | --- | --- | --- |
| Concentration | 1 | 0.63 | 0.63 | 1531.27 | 1.9e-09 |  |
| Type (*standard or pooled sample*) | 1 | 0.0009 | 0.0009 | 2.17 | 0.19 |  |
| Concentration*Type | 1 | 0.001 | 0.001 | 3.51 | 0.10 |  |
| Residuals | 7 | 0.003 | 0.0004 |  |  |  |
|  |  |  |  |  |  |  |

|  |  |  |  |  |  |
| --- | --- | --- | --- | --- | --- |

**Table S2. Generalized linear mixed models of baseline corticosterone for *R. catesbeiana* (A), *R. clamitans* (B), and *R. pipiens* (C).** **Significant terms are bolded.**

*A. Rana catesbeiana*

| Coefficient | Estimate | S.E. | df | t-value | p-value | |
| --- | --- | --- | --- | --- | --- | --- |
| **(Intercept)** | **-0.91** | **0.20** | **27** | **-4.68** | **7.3e-5** | |
| Sex (M) | 0.10 | 0.35 | 25 | 0.30 | 0.76 | |
| Body Condition | -0.07 | 0.16 | 27 | -0.43 | 0.67 | |
|  |  |  |  |  |  | |
| Random effect | Variance | S.D. |  |  |  |  |
| ID | 0.0 | 0.0 |  |  |  |  |
|  |  |  |  |  | |  |

*B. Rana clamitans*

| Coefficient | Estimate | S.E. | df | t-value | | | p-value |
| --- | --- | --- | --- | --- | --- | --- | --- |
| **(Intercept)** | **-1.00** | **0.16** | **28** | **-6.27** | | | **9e-7** |
| Sex (M) | 0.28 | 025 | 28 | 1.11 | | | 0.28 |
| Body Condition | -0.07 | 0.13 | 28 | -0.59 | | | 0.56 |
|  |  |  |  |  | | |  |
| Random effect | Variance | S.D. |  |  |  |  |  |
| ID | 0.0 | 0.0 |  |  |  |  |  |
|  |  |  |  | |  |  |  |

*C. Rana pipiens*

| Coefficient | Estimate | S.E. | df | t-value | | p-value |
| --- | --- | --- | --- | --- | --- | --- |
| **(Intercept)** | **-1.51** | **0.20** | **15.0** | **-7.57** | | **1.7e-6** |
| **Sex** | **0.98** | **0.29** | **15.2** | **3.36** | | **0.004** |
| Body Condition | 0.07 | 0.11 | 17.2 | 0.64 | | 0.53 |
|  |  |  |  |  | |  |
| Random effect | Variance | S.D. |  | |  |  |
| ID | 0.12 | 0.35 |  |  |  |  |
